# Impact of Nisin on Growth Inhibition and Biofilm Formation Capacity of *Listeria monocytogenes*

**DOI:** 10.3390/ijms27167355

**Published:** 2026-08-17

**Authors:** Sandra Smoter, Joanna Gajewska, Magdalena A. Olszewska

**Affiliations:** Department of Food Microbiology, Meat Technology and Chemistry, The Faculty of Food Science, University of Warmia and Mazury in Olsztyn, Plac Cieszyński 1, 10-726 Olsztyn, Poland

**Keywords:** *Listeria monocytogenes*, nisin, growth inhibition, biofilm formation, biofilm-related gene expression

## Abstract

The effects of nisin on growth inhibition and biofilm formation were studied in two *Listeria monocytogenes* strains, LM_ATCC 19115 and LM_18, at concentrations ranging from 14 to 450 µg/mL. The maximum rate of OD_600_ increase (μ_max_) showed no significant concentration-dependent change in LM_ATCC 19115, whereas LM_18 exhibited a downward trend. Maximum OD_600_ dropped progressively and significantly at 225 and 450 µg/mL in both strains. Although concentrations of 14–56 µg/mL supported gradual growth that paralleled the control, the highest nisin concentrations significantly prolonged the lag phase of both strains and eventually decreased biomass by 64% and 51% for LM_ATCC 19115 and LM_18, respectively. Consequently, nisin reduced biofilm formation, with no biofilm observed for LM_ATCC 19115 at 450 µg/mL. However, both strains retained biofilm-forming capacity at 14–113 µg/mL concentrations. In LM_ATCC 19115, biofilm formation decreased independently of growth, along with significantly increased expression of the motility gene *flaA* and quorum sensing regulator *agrA*. In contrast, for LM_18, biofilm reduction corresponded to growth inhibition, with significant downregulation of the virulence gene *hly*. The observed strain-specific patterns of growth and biofilm formation warrant further investigation with a broader range of strains to better define and address the limitations of nisin application.

## 1. Introduction

Foodborne illnesses caused by bacterial pathogens are an increasing global public health concern [1]. One of the most significant pathogens is *Listeria monocytogenes* (LM), which often persists in food processing environments [2]. Several theories explain the persistence of LM, including biofilm formation, which enhances resistance to environmental stress and antimicrobial treatments [3]. In fact, biofilm formation allows LM to persist on food-contact surfaces despite routine cleaning and sanitation efforts [4]. The development of *L. monocytogenes* biofilms is influenced by environmental conditions, such as temperature and nutrient availability, as well as internal regulatory mechanisms, such as strain serotype, extracellular polymeric substance production, and quorum sensing (QS) systems [5].

Due to the significant public health and economic burden associated with *L. monocytogenes*, researchers and the food industry continue to explore effective strategies to control this pathogen. Among these, biopreservation using natural antimicrobial compounds has attracted considerable attention. Nisin is a well-known bacteriocin produced by *Lactococcus lactis* subsp. *lactis*, classified as “Generally Recognized as Safe” (GRAS) by the US Food and Drug Administration [6,7]. Owing to its potent activity against Gram-positive bacteria, including *L. monocytogenes*, nisin has been widely adopted as a natural food preservative [8].

While there are several studies on variations in nisin resistance among foodborne *L. monocytogenes* [5,9,10], less is known about how nisin affects biofilm formation in *L. monocytogenes* and whether that effect also varies strain-by-strain. The main objective of this study was to investigate the effects of nisin on the growth and biofilm formation of two strains of *L. monocytogenes*. One strain is a well-known reference strain classified as serotype 4b (ATCC 19115), while the other is a food processing environment isolate deposited in our collection, classified as serotype 1/2b by PCR. These strains were selected for their strong capacity to form biofilms, as identified during a preliminary screening. We also examined selected genes associated with virulence (*hly*) and motility (*flaA*) and *quorum-sensing* (*agrA*) in *L. monocytogenes* to better understand the factors contributing to the survival and differing biofilm-forming capacities of the tested strains. This study aims to provide a clearer understanding of the potential risks posed by foodborne *L. monocytogenes* and the implications of using biopreservatives in food products.

## 2. Results

Both strains, LM_ATCC 19115 and LM_18, had a nisin MIC of 125 µg/mL. Their growth inhibition was tested at nisin concentrations from 14 µg/mL to 450 µg/mL, analyzed using the modified Gompertz model.

### 2.1. Growth Inhibition of LM Strains

**Figure 1:**
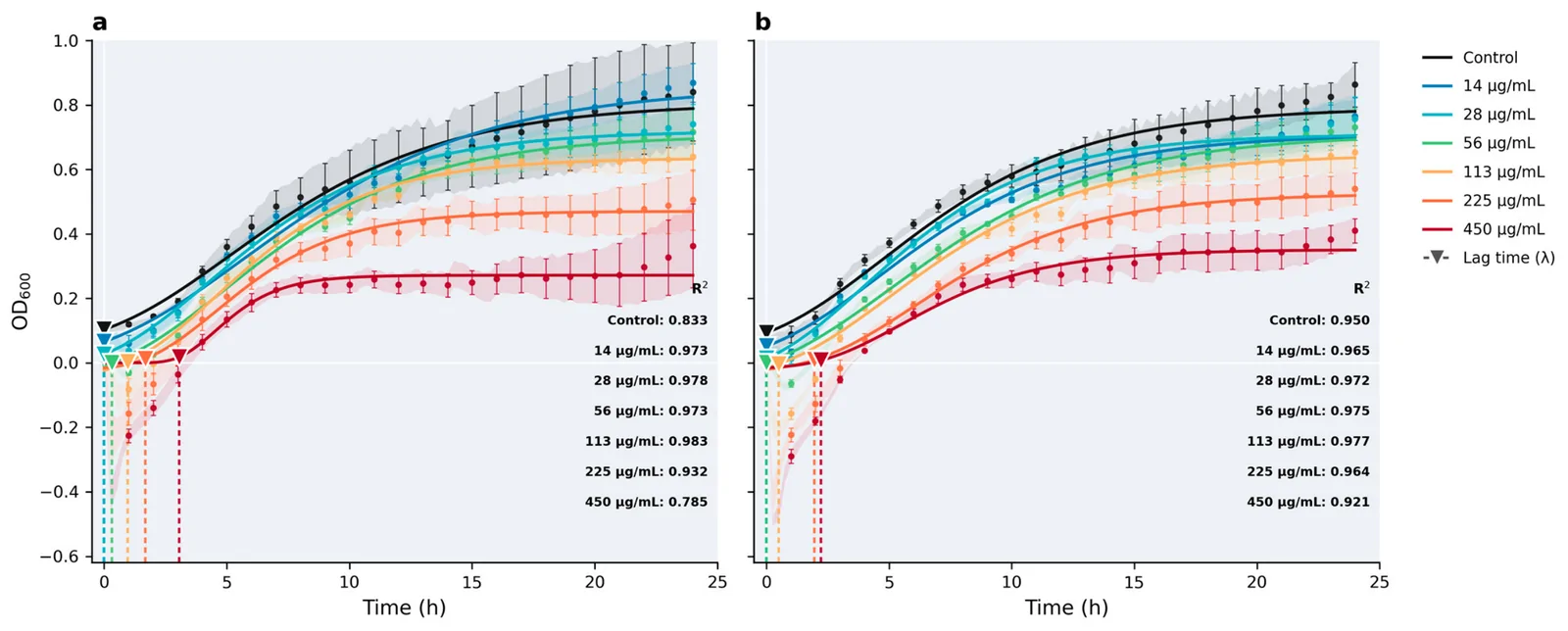
Fitted modified Gompertz growth curves for the two *L. monocytogenes* strains: LM_ATCC 19115 (**a**) and LM_18 (**b**) across the untreated control and six nisin concentrations (mean ± SD shown as a shaded envelope). Downward triangles and dashed guide lines mark the fitted lag time (λ) for each concentration; exact λ values are reported in Table 1.

Fitted modified Gompertz growth curves for the two *L. monocytogenes* strains are shown in Figure 1. The strain LM_ATCC 19115 achieved a maximum optical density at 600 nm (OD_600_) of 0.81, with a maximum rate of OD_600_ increase (μ_max_) of 0.054 h^−1^. Meanwhile, strain LM_18 reached a maximum OD_600_ of 0.79, with a μ_max_ of 0.058 h^−1^ (Table 1). Model fit was at R^2^ ≥ 0.92 across both strains and nisin concentrations, with two exceptions in LM_ATCC 19115: the control (R^2^ = 0.83) and the 450 µg/mL nisin (R^2^ = 0.79) (Figure 1).

For LM_18, the lag time increased with nisin concentration, reaching 0.50 h at 113 µg/mL, 1.96 h at 225 µg/mL (*p* < 0.0001), and 2.23 h at 450 µg/mL (*p* < 0.0001) (Table 1). Lag time for LM_ATCC 19115 also increased with concentration, starting at 0.32 h at 56 µg/mL and extending to 0.96 h, then 1.67 h, and finally reaching 3.05 h at 450 µg/mL, which was longer than LM_18’s lag time of 2.23 h at the same highest concentration.

Both strains exhibited a loss of biomass as nisin concentration increased, with minimal effect noted below 113 µg/mL. At the highest concentration, LM_ATCC 19115 showed a greater relative loss of biomass compared to LM_18 (64% inhibition versus 51%; *p* < 0.0001). This may indicate that LM_ATCC 19115 cells are more susceptible, as nisin considerably affected final yield, with significant differences from the control observed at both 225 and 450 µg/mL.

In terms of maximum rate of OD_600_ increase, this parameter was the least responsive in both strains. Only LM_18 showed the expected effect at the highest concentration, 450 µg/mL, resulting in 29% inhibition (*p* = 0.0014). In contrast, LM_ATCC 19115 showed no consistent effect on absolute growth rate (Table 1). Notably, Supplementary Table S1 indicates whether the nisin concentration had an overall effect on each growth parameter for each strain across all nisin concentrations. In both strains, λ and Max OD_600_ showed significant effects, whereas μ_max_ was significant only in LM_18 (*p* = 0.0001).

**Figure 2:**
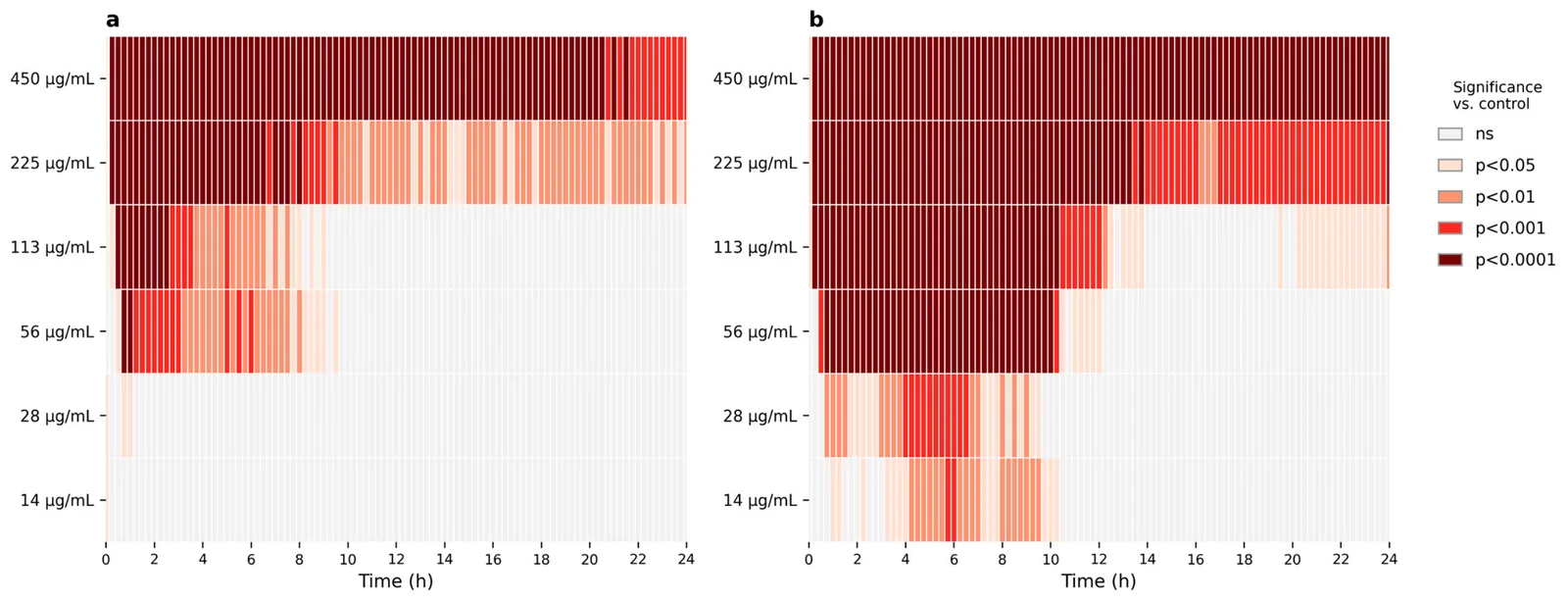
Heatmap showing the time-resolved significance of each treatment compared to control in the two strains of *L. monocytogenes*: LM_ATCC 19115 (**a**) and LM_18 (**b**), analyzed using one-way ANOVA followed by Tukey’s HSD post hoc test at every time point (n = 3 for each group within the 7-group test, uncorrected across time points).

The time-resolved analysis presented in Figure 2 shows how the statistical significance of each treatment differs from the untreated control as nisin concentration increases. For LM_18, the proportion of time points reaching significance increased from 33% at 14 µg/mL, 37% at 28 µg/mL, 47% at 56 µg/mL, and 74% at 113 µg/mL, before reaching 100% at both 225 and 450 µg/mL (Supplementary Table S2). LM_ATCC 19115 followed a similar but less consistent trend across the same six concentrations: 1%, 3%, 37%, and 35% at 14, 28, 56, and 113 µg/mL, respectively, rising to 99% at both 225 and 450 µg/mL.

### 2.2. Biofilm Formation Capacity of LM Strains

**Figure 3:**
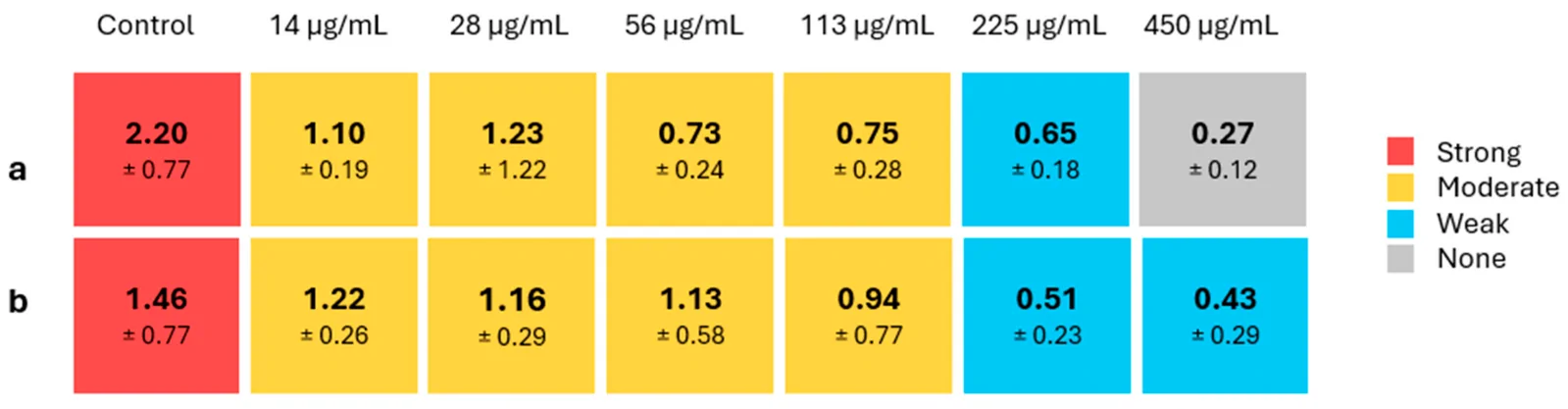
Biofilm-forming ability of the two strains of *L. monocytogenes*: LM_ATCC 19115 (**a**) and LM_18 (**b**) in the presence of increasing nisin concentrations. Each cell displays the mean optical density (OD_570_) ± standard deviation. Cell color indicates the biofilm-forming classification: gray—no biofilm (OD ≤ ODc); blue—weak (ODc < OD ≤ 2 × ODc); amber—moderate (2 × ODc < OD ≤ 4 × ODc); red—strong (OD > 4 × ODc), where ODc represents the optical density of the negative control corrected by three standard deviations.

Strains exhibited varying baseline optical density (OD_570_) readings. LM_ATCC 19115 showed an OD_570_ of 2.2 ± 0.76, while LM_18 recorded an OD_570_ of 1.46 ± 0.53. Both strains were classified as strong, as illustrated in Figure 3. They both maintained a moderate classification at concentrations from 14 µg/mL to 113 µg/mL. The key difference appears at the higher concentrations. LM_18 reached a weak classification at 225 and 450 µg/mL, with values of 0.51 and 0.43, respectively. In contrast, LM_ATCC 19115 decreased to none at the 450 µg/mL concentration.

For LM_ATCC 19115, the specific biofilm formation (SBF; Table 2) index declines to 36–63% of the control across all tested concentrations, including at 14 µg/mL, where growth is not inhibited (106% of control). Despite this, the SBF index still drops to 47% of control, suggesting a growth-independent antibiofilm effect, since there is no reduction in bacterial biomass.

In contrast, for LM_18, the SBF index aligns more closely with the reduction in raw growth, particularly at low to moderate concentrations (14–113 µg/mL). Here, the SBF index ranges from 79% to 93%, while growth decreases to between 82% and 90%. This indicates that most of the biofilm reduction may be due to reduced bacterial biomass. At higher concentrations, a modest independent effect is observed at 225 µg/mL (SBF 53% vs. growth 66%). However, at 450 µg/mL, the SBF index recovers to 67%, while growth remains at 44%.

### 2.3. Virulence and Biofilm-Associated Gene Expression

**Figure 4:**
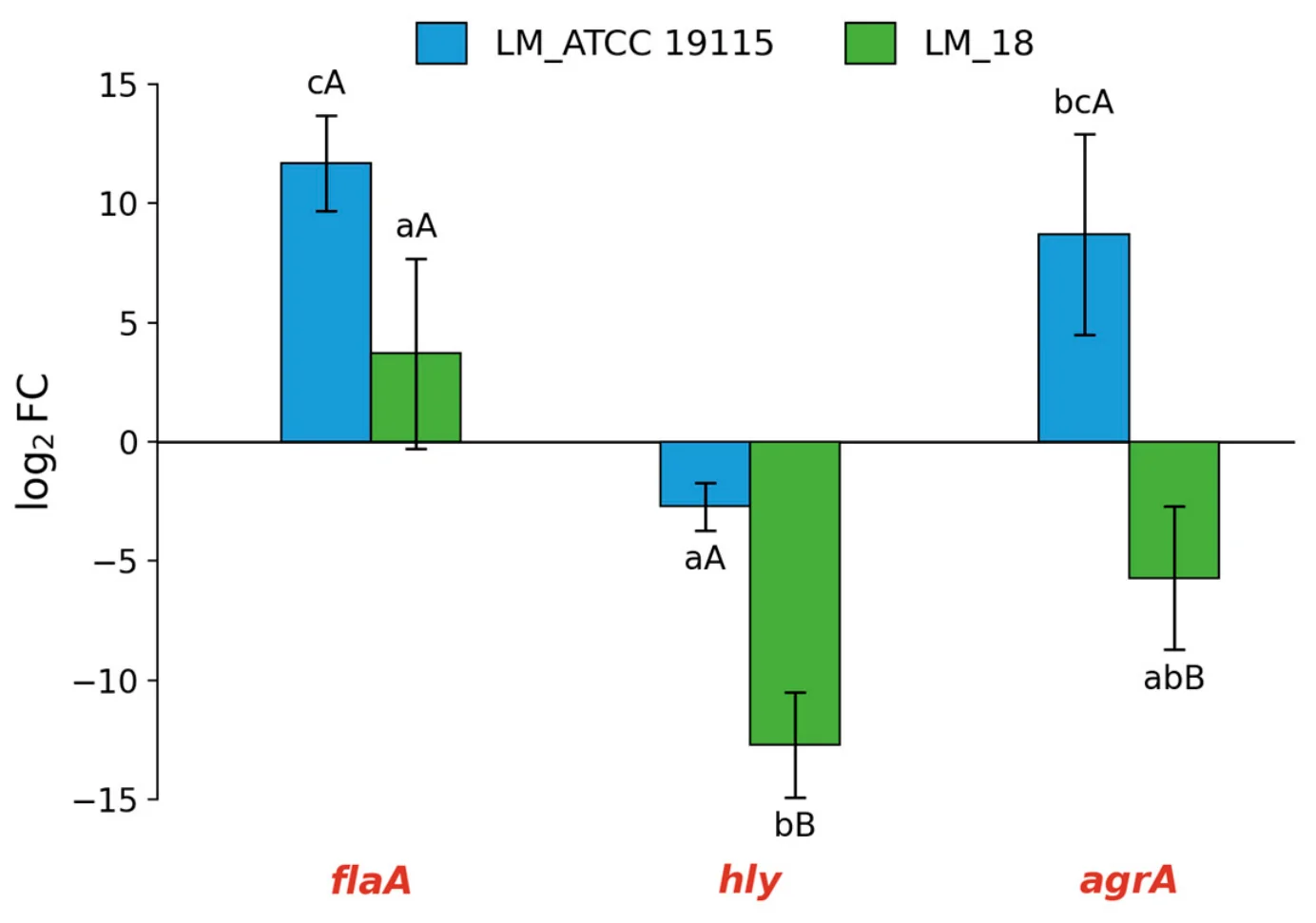
Effect of a sublethal nisin concentration (56 µg/mL) on the relative expression of *flaA*, *hly* and *agrA* in *L. monocytogenes* strains: LM_ATCC 19115 and LM_18. Data are presented as mean log_2_ fold change (log_2_FC) ± SD. Different lowercase letters (a–c) indicate statistically significant differences among genes within the same strain (*p* < 0.05). Different uppercase letters (A,B) indicate statistically significant differences between strains within a given gene (*p* < 0.05). *flaA*—flagellin A; *hly*—listeriolysin O; *agrA*—accessory gene regulator A.

Exposure to nisin at 56 µg/mL induced strain-dependent expression changes in the target genes (Figure 4).

LM_ATCC 19115 exhibited significant upregulation of the *flaA* and *agrA* genes, with log_2_FC values of 11.6 and 8.7, respectively, compared to the *hly* gene (Figure 4), and both compared to the control (Supplementary Table S3). LM_18 showed moderate *flaA* upregulation and *agrA* downregulation, with a log_2_FC of −5.5, differing from LM_ATCC 19115. While LM_ATCC 19115 displayed only a slight decrease in *hly* expression, LM_18 exhibited pronounced downregulation, reaching a log_2_FC of −11.9, which was also significant when compared to the control.

## 3. Discussion

This study investigated the effect of nisin on the growth and biofilm formation of two *Listeria monocytogenes* strains. Nisin exerted inhibitory effects, with strain variability between *L. monocytogenes* ATCC 19115 and a food processing isolate, LM_18, reflected in prolonged lag phases of 3.05 and 2.23 h and biomass reductions of 64% and 51%, respectively (Figure 1/Table 1). Lower concentrations still supported gradual growth, which was largely paralleled with the control at 14–28 µg/mL (Figure 2). This pattern was mirrored in biofilm formation, which was abolished entirely for LM_ATCC 19115 at 450 µg/mL, though both strains still formed biofilms at concentrations between 14 and 113 µg/mL (Figure 3). Nisin is well known to be inhibitory to a wide range of Gram-positive bacteria and acts in two ways, firstly by forming pores in the bacterial cell membrane, and secondly by inhibiting peptidoglycan synthesis [11]. Although effective against *L. monocytogenes*, its activity is strain- and environmental condition-specific, which translates into different reported MIC values, ranging from 1.5 µg/mL [12] to 320 µg/mL [5], as well as patterns of growth and biofilm formation. Previous research [5] demonstrated that nisin at 320 µg/mL delayed the growth of *L. monocytogenes* ATCC 19114 and CMCC 8605, with lag phases extended by 4 h and 6 h, respectively, based on OD_600_, before ultimately reaching a maximum OD of 0.4 in both strains. Interestingly, the time-kill curve showed that log CFU decreased from 9 log CFU/mL to 7.5 and 7 log CFU/mL, respectively, after only 1 h of incubation with nisin, and further decreased to 5 and 4 log CFU/mL after 6 h, due to viable-but-non-culturable (VBNC) phenomena affecting culturability independent of OD-based growth measures. Similarly, Yan et al. [13] reported that nisin at 100 μg/mL resulted in growth inhibition of 1.1 and 0.3 log CFU/mL for *L. monocytogenes* ATCC 7644 and the food-isolated MRL 300091 after 3 h incubation. However, as the treatment time was extended, nisin did not exhibit a significant inhibitory effect on growth. Liu et al. [14] studied the survival of *L. monocytogenes* Scott A in the presence of nisin concentrations of 5–150 µg/mL and reported no reduction in CFU for 24 h. Wu et al. [12] monitored the death kinetics of *L. monocytogenes* NCTC 7973 and a food-environment isolate, A1, at concentrations up to 50 times the MIC. Cell counts of strain A1 decreased at a nisin concentration 8 times the MIC (12 µg/mL), while cell counts of strain NCTC 7973 decreased at a nisin concentration 12 times the MIC (18 µg/mL), with a population of persister cells observed at higher nisin concentrations. Moreover, nisin at 320 µg/mL also exerted an inhibitory effect on biofilm production, resulting in decreased biofilm formation rates of 80% and over 80% in CMCC 8605 and ATCC 19114, respectively [5]. In contrast, Yan et al. [13] reported that, compared with the control group, nisin at 100 µg/mL did not significantly reduce biofilm formation in strains ATCC 7644 and 300091 at 24, 48, or 72 h based on plate counting, nor did it change the three-dimensional structure or the amount of extracellular polysaccharides.

Furthermore, we also investigated the effect of nisin at 56 µg/mL, a concentration at which no biomass inhibition was observed for either strain, on the transcript levels of *flaA* (associated with flagellar structure), *agrA* (main regulator of the QS system), and *hly* (encoding the primary protein toxin, listeriolysin O). In LM_ATCC 19115, although growth was not significantly reduced at this concentration, biofilm-forming capacity, normalized to the amount of growth achieved, fell to 38% of control, implying an antibiofilm effect independent of growth inhibition. It is important to note that the crystal violet assay stains the entire biofilm, including both attached cells and the extracellular polymeric substance (EPS) matrix, and our biofilm assay results show pronounced reductions starting at 14 µg/mL in LM_ATCC 19115, with no biofilm remaining at the highest concentration. At 56 µg/mL in LM_ATCC 19115, significant upregulation of the genes *flaA* and *agrA* was observed. In contrast, strain LM_18 exhibited growth at 90% of the control, with the SBF index at 86% of the control, consistent with biofilm reduction here being driven mainly by reduced growth. As for the two genes, *flaA* and *agrA*, they were upregulated and downregulated, respectively, in LM_18. The *flaA* gene encodes flagellin A, the primary structural protein of the flagellum, important for initial surface attachment and early biofilm development [15]. As part of *agrA*’s role in QS, the peptide-mediated QS signaling system Agr is associated with the formation of *L. monocytogenes* biofilms [16]. It has been reported that *agrA* gene-deficient strains have a significantly reduced ability to form biofilms compared with wild-type strains, and that natural antimicrobials can reduce biofilm formation by acting on the bacterial group-sensing system, including inhibition of *agrA* expression [13]. Yu et al. [5] have shown that nisin significantly downregulated the expression levels of *agrA* and other *agr* genes in *L. monocytogenes* CMCC 8605, but not in the other strain, ATCC 19114. Not all nisin tests confirm inhibition of the QS system. Consistent with our findings, Yan et al. [13] reported that nisin increased the expression of *agrA* in ATCC 7644 at a subinhibitory 50 µg/mL (37 °C/6 h), but not in a food isolate, MRL 300091. As a hypothesis, we propose that the antimicrobial/anti-biofilm activity of nisin at least partially originates from blocking the Agr system in LM_18, but not in LM_ATCC 19115, where *agrA* was significantly up-regulated. It is also possible that the energetic cost of arginine biosynthesis, a metabolically expensive pathway in which *argA* is involved [5], competes with, and thereby limits, the energy available for biofilm formation in strains overexpressing this gene.

Although Yan et al. [13] did not investigate *flaA*, they reported that *fbpB*, another surface-associated gene, was significantly up-regulated, with expression levels differing markedly between the two strains tested (ATCC 7644 and MRL 300091). The *fbpB* gene encodes a fibronectin-binding protein, whose expression changes in response to temperature and nutrient stress when *L. monocytogenes* transitions into a biofilm state [17]. As for *flaA*, Stincone et al. [15] demonstrated that proteins associated with biofilm formation in *L. monocytogenes*, including flagellin-related proteins, were among the surface proteins upregulated following nisin exposure. To validate the proteomic findings, increased biofilm formation in nisin-treated cells was subsequently confirmed experimentally using the crystal violet assay, with nisin applied at 0.1 μg/mL and incubation performed at 37 °C. *L. monocytogenes* is highly flagellated and motile at temperatures below 37 °C; therefore, motility-related genes, including *flaA*, are generally downregulated at higher temperatures [18]. However, temperature-dependent control of motility gene expression may not be equally stringent under all conditions. For example, Gründling et al. [19] reported that MogR-mediated repression of *flaA* was independent of temperature during intracellular infection. Similarly, Kragh and Truelstrup Hansen [18] demonstrated that *L. monocytogenes* 08-5578 downregulated 28 motility-related genes, including *flaA*, during desiccation stress, despite simultaneous downregulation of *mogR*, which encodes the motility repressor. Nonetheless, less motile strains appear to adapt more rapidly to stress than highly motile strains [20]. Similarly, Cabrita et al. [21] observed significant differences in relative gene expression of *flaA* between a persistent and presumed nonpersistent strain, with lower expression in the persistent strain, suggesting that gene expression networks are differently adjusted in the two strains to the low-temperature environment from where they were collected. A reduction or loss of motility appears to be advantageous for stress survival, possibly because it reduces the energetic investment in these processes. Although *flaA* in LM_18 was upregulated, the increase was not statistically significant relative to the control, unlike in the ATCC_19115 strain. Thus, as a hypothesis, we suggest that the environmental isolate may be better adapted to survive nisin stress, assuming that nisin would actually affect motility.

Moreover, LM_18 showed significantly reduced *hly* expression, which may reflect reduced investment in virulence-associated functions during nisin stress. Given its isolation source from a food-processing environment, this response may indicate adaptation to environmental stress. Interestingly, Babakhani et al. [22] reported that nisin treatment (100 IU/mL, 37 °C, 24 h) reduced *flaA* expression and the invasion ability of a sausage isolate of *L. monocytogenes*. Moreover, nisin solutions at 100, 1000, and 10,000 IU/mL inhibited biofilm formation by 99%, 90%, and 56%, respectively. Li et al. [23] reported that *hly* deletion in LM 10403S significantly impaired biofilm formation, where the Δ*hly* strain had reduced biofilm biomass and a looser structure than wild-type and substantially less EPS in the mutant. Indeed, previous research has shown that exposure to nisin represses virulence genes, including *hly* and the ability to form biofilms [13,24,25]. However, whether this was a critical factor in decreasing biofilm formation should be interpreted with caution, and further validation is required to confirm this correlation. Further proteomic investigations are required to determine whether these changes are reflected at the protein level and ultimately translate into corresponding phenotypic responses. Moreover, an important limitation of the present study is that only two *L. monocytogenes* strains were examined. Consequently, the results should be considered strain-specific rather than representative of the species, and confirmation in a larger, genetically diverse strain collection will be necessary.

## 4. Materials and Methods

### 4.1. Bacterial Strains

Two strains of *Listeria monocytogenes* (LM): ATCC 19115 and 18 were used in this study and were obtained from our culture collection at the Department of Food Microbiology, Meat Technology and Chemistry, University of Warmia and Mazury in Olsztyn, Poland. Stock cultures were activated by transferring each strain to tryptic soy agar (TSA; Merck, Darmstadt, Germany) and incubating at 37 °C for 24 h. Working cultures were prepared through two transfers into tryptic soy broth (TSB, Merck) followed by overnight incubation at 37 °C.

### 4.2. Biopreservative

Nisin from *Lactococcus lactis* (CAS No: 1414-45-5) was obtained from Pol-Aura (Olsztyn, Poland).

### 4.3. Minimum Inhibitory Concentration (MIC)

To determine the minimum inhibitory concentration (MIC) of nisin, we performed a broth microdilution assay using 96-well microtiter plates (Sarstedt, Nümbrecht, Germany) as per CLSI standards. Each well contained a bacterial culture in TSB at approximately 10^5^ CFU/mL and nisin solutions at concentrations ranging from 3.91 µg/mL to 1000 μg/mL, with a total volume of 200 μL. After incubation at 37 °C for 24 h, the MIC was determined as the lowest concentration inhibiting bacterial growth, measured by optical density at 620 nm (OD_620_) using the Multiskan™ GO plate reader (Thermo Fisher, Waltham, MA, USA).

### 4.4. Bacterial Growth

The effects of nisin on LM strains were analyzed in TSB by measuring turbidity resulting from bacterial growth, as described by Sudagidan and Yemenicioğlu [26]. Strains were grown overnight on TSA at 37 °C, then suspended in 10 mL of 0.9% sodium chloride (NaCl) and adjusted to a McFarland standard of 0.5. In a 96-well plate, 180 µL of TSB containing nisin at prepared concentrations of 16 to 500 µg/mL was combined with 20 µL of the test strain suspension (total volume 200 µL), giving final nisin concentrations of 14.1, 28.1, 56.3, 112.5, 225.0, and 450.0 µg/mL (rounded to 14, 28, 56, 113, 225, and 450 µg/mL). The plate was incubated at 37 °C for 24 h in a microplate reader (Varioskan LUX, Thermo Fisher, Waltham, MA, USA), and absorbance was measured at 600 nm every 15 min. Controls and nisin cultures were tested in triplicate wells, and the original measured values were retained for the mean ± SD growth-curve plots. Growth curves were fitted with the modified Gompertz equation reported by Zwietering et al. [27]:

(1) $$OD\left(t\right)\;=\;OD_{0}\;+\;A\;\cdot \;\exp\left\{-\exp\left[\frac{\mu_{max}\;\cdot \;e}{A}\left(\lambda \;-\;t\right)\;+\;1\right]\right\}$$

where OD_0_ is the baseline (pre-growth) absorbance, A is the maximum increase in OD_600_ above baseline (i.e., asymptotic net biomass gain), μ_max_ is the maximum absolute growth rate (the slope of the tangent at the inflection point, OD_600_ h^−1^), λ is the lag time (h; the x-intercept of that tangent), t is time (h), and e is Euler’s number. Maximum OD_600_ is reported as OD_0_ + A. Goodness of fit is reported as R^2^. Percent inhibition relative to the untreated control was calculated for the growth rate:

(2) $$\%\;Inhibition_{\mu max}\;=\;100\;\times \;\left(1\;-\;\frac{\mu_{max,\;treatment}}{\mu_{max,\;control}}\right)$$

and for the net biomass yield:

(3) $$\%\;Inhibition_{A}\;=\;100\;\times \;\left(1\;-\;\frac{A_{treatment}}{A_{control}}\right)$$

Positive values indicate inhibition, and negative values indicate a value above the control.

For each treatment, we also compared it directly to its corresponding strain-matched untreated control at 97 sampled time points (0–24 h, with a 15 min resolution). This complementary, model-free analysis utilized raw (blank-subtracted) OD_600_ measurements and allowed us to visualize when each treatment diverged from the untreated control throughout the 24 h time course.

### 4.5. Biofilm Formation Assay

After incubation, the growth inhibition test plates were evaluated for biofilm formation. The plates were washed five times with phosphate-buffered saline (PBS, pH 7.0; Sigma-Aldrich, St. Louise, MO, USA), then fixed with ethanol for 15 min and dried at 55 °C for 1 h. Next, the biofilm was stained with crystal violet for 5 min, and excess dye was removed with water. After drying, the crystal violet was dissolved in 33% glacial acetic acid, and the optical densities were measured at 570 nm using the Multiskan GO microplate reader. The biofilm-forming potential of the strains was classified according to the criteria proposed by Stepanović et al. [28]. Strains were categorized as strong biofilm producers when the optical density (OD) was greater than four times the cut-off optical density (ODc), moderate biofilm producers when 2 × ODc < OD ≤ 4 × ODc, weak biofilm producers when ODc < OD ≤ 2 × ODc, and non-biofilm producers when OD ≤ ODc.

Because growth (OD_600_, 24 h) and biofilm (OD_570_, crystal violet) were measured in the same wells, OD_600_ was recorded after 24 h of growth in TSB, after which the same wells were washed and stained, and a specific biofilm formation (SBF) index (OD_570_/OD_600_) was calculated to differentiate between growth inhibition and antibiofilm effects.

### 4.6. RNA Extraction and Real-Time PCR

The effects of nisin on the expression of *flaA*, *hly*, and *agrA* genes were studied using real-time PCR [29] with a RotorGene Q System (Qiagen Inc., Montreal, ON, Canada). Total RNA was extracted from control samples and those treated with 1/2 × MIC after 6 h incubation at 37 °C in TSB, corresponding to the exponential growth phase and consistent with the antimicrobial exposure conditions used in the expression of LM genes [30], using the Total RNA Mini Plus Kit (A&A Biotechnology, Gdańsk, Poland). RNA was purified with the Clean-Up RNA Concentrator Kit. First-strand cDNA synthesis was performed using the TranScriba Kit (A&A Biotechnology) and a dN-hexamer primer, and the cDNA was stored at −20 °C for analysis. Three independent reactions were conducted for each gene, and amplification efficiency was calculated from the slope of the primer pair data (Supplementary Table S4). Gene expression was assessed using the comparative critical Ct method, with results expressed as fold changes relative to the control group.

### 4.7. Statistical Analysis

To test for statistically significant differences between treatments, the modified Gompertz model was fitted separately to each of the three biological replicates, yielding an independent estimate of μ_max_, λ, and maximum OD_600_ for every strain × treatment × replicate combination. For each strain and each parameter (μ_max_, λ, maximum OD_600_), a one-way ANOVA was first run across all seven treatments to test for an overall treatment effect. Then, Dunnett’s multiple-comparison test was used to compare each antimicrobial concentration against the strain’s untreated control.

The time-resolved comparison and gene expression comparison were performed by running a one-way ANOVA across all seven groups (control + six treatments, n = 3 each) at each time point, then extracting the treatment-vs-control pairwise result from Tukey’s post hoc test. Significance is denoted throughout as * *p* < 0.05, ** *p* < 0.01, *** *p* < 0.001, **** *p* < 0.0001, and “ns” for not significant (*p* ≥ 0.05). Statistical analyses were performed in using SciPy and NumPy in Python (version 3.12) and TIBCO^®^ Statistica™ ver. 13.3 (TIBCO Software Inc., Tulsa, OK, USA) for nonlinear regression, ANOVA, and post hoc testing.

## 5. Conclusions

This study demonstrates that nisin reduced final biomass yield and biofilm formation of the tested *Listeria monocytogenes* strains, with significant effects observed at 225 and 450 µg/mL. In LM_ATCC 19115, nisin’s primary impact was on lag phase and final population density rather than exponential-phase growth kinetics, whereas LM_18 also showed a significant reduction in growth rate at the highest concentration tested (450 µg/mL). Importantly, when biofilm biomass was normalized to bacterial biomass yield, the antibiofilm effect was not solely a secondary consequence of reduced growth. In strain LM_ATCC 19115, the capacity to form biofilm decreased independently of biomass yield. In contrast, for LM_18, the reduction in biofilm formation was more closely associated with reduced biomass yield. This phenotypic distinction was also observed at the gene expression level: LM_ATCC 19115 exhibited up-regulation of *flaA* and *agrA*, indicating a biomass-independent stress response, whereas LM_18’s response was limited to *hly* and was more directly linked to the suppression of biomass accumulation. As *L. monocytogenes* exhibited strain-dependent patterns of growth and biofilm formation, with adaptive traits potentially emerging under nisin stress, our findings highlight the need for further research into time- and concentration-dependent gene expression responses, detailed characterization of sessile-cell structures, and evaluation of nisin in combination with other antimicrobials using a larger and more genetically diverse strain collection, to better define and address the limitations of nisin application.

## Figures and Tables

**Table 1:** Modified Gompertz growth parameters and percent inhibition relative to the untreated control for the two *L. monocytogenes* strains exposed to different nisin concentrations.

Strain	Nisin Concentration	μ_max_ (h^−1^)	λ (h)	Max OD_600_	μ_max_ Inhibition (%)	Biomass Inhibition (%)
LM_ATCC 19115	Control	0.054 ± 0.003	0.00 ± 1.46	0.807	–	–
	14 µg/mL	0.055 ± 0.001	0.00 ± 0.61	0.857	−1.0	−12.6
	28 µg/mL	0.064 ± 0.001	0.00 ± 0.40	0.720	−17.6	1.4
	56 µg/mL	0.060 ± 0.001	0.32 ± 0.46	0.707	−10.5	−0.5
	113 µg/mL	0.069 ± 0.001	0.96 ± 0.24	0.635	−27.4	10.0
	225 µg/mL	0.062 ± 0.003	1.67 ± 0.34	0.470 **	−14.3	35.0
	450 µg/mL	0.067 ± 0.008	3.05 ± 0.32 *	0.272 ****	−22.6	63.9
LM_18	Control	0.058 ± 0.002	0.00 ± 0.69	0.792	–	–
	14 µg/mL	0.057 ± 0.001	0.00 ± 0.55	0.709	2.1	6.0
	28 µg/mL	0.069 ± 0.002 *	0.00 ± 0.43	0.709	−17.4	0.5
	56 µg/mL	0.055 ± 0.001	0.00 ± 0.52	0.711	5.3	−1.6
	113 µg/mL	0.056 ± 0.001	0.50 ± 0.40	0.647	3.8	6.9
	225 µg/mL	0.050 ± 0.001	1.96 ± 0.34 ****	0.526 **	14.8	26.2
	450 µg/mL	0.041 ± 0.002 **	2.23 ± 0.38 ****	0.351 ****	29.1	50.9

μ_max_: maximum absolute growth rate; λ: lag time; Max OD_600_: maximum optical density at 600 nm (OD_0_ + A). Inhibition (%) = 100 × (1 − treatment/control); negative values indicate a parameter value exceeding the untreated control; – not applicable (reference group). Data are parameter estimates from nonlinear least-squares fitting of the modified Gompertz equation per strain and treatment. Asterisks denote a statistically significant difference from the untreated control (one-way ANOVA with Dunnett’s post hoc test, fitted to each replicate individually): * *p* < 0.05, ** *p* < 0.01, **** *p* < 0.0001.

**Table 2:** Specific Biofilm Formation (SBF) index by strain and treatment, with OD_570_ and OD_600_ values used to calculate biofilm and growth percentage of each *L. monocytogenes* strain.

Strain	Nisin Concentration	OD_570_	OD_600_	Biofilm (% Ctrl)	Growth (% Ctrl)	SBF Index (% Ctrl)
LM_ATCC 19115	Control	2.20	0.81	100%	100%	100%
	14 µg/mL	1.10	0.86	50%	106%	47%
	28 µg/mL	1.23	0.72	56%	89%	63%
	56 µg/mL	0.73	0.71	33%	88%	38%
	113 µg/mL	0.75	0.63	34%	79%	43%
	225 µg/mL	0.65	0.47	30%	58%	51%
	450 µg/mL	0.27	0.27	12%	34%	36%
LM_18	Control	1.46	0.79	100%	100%	100%
	14 µg/mL	1.22	0.71	84%	90%	93%
	28 µg/mL	1.16	0.71	80%	90%	89%
	56 µg/mL	1.13	0.71	77%	90%	86%
	113 µg/mL	0.94	0.65	64%	82%	79%
	225 µg/mL	0.51	0.53	35%	66%	53%
	450 µg/mL	0.43	0.35	30%	44%	66%

SBF index = OD_570_/OD_600_, computed per well; “% ctrl” columns express each value as a percentage of the strain’s own untreated control.

## Data Availability

The data that support the findings of this study are openly available in Zenodo at https://doi.org/10.5281/zenodo.21177461.

## Supplementary Materials

The following supporting information can be downloaded at: https://www.mdpi.com/article/10.3390/ijms27167355/s1.

## Abbreviations

The following abbreviations are used in this manuscript:

ANOVA	Analysis of Variance
ATCC	American Type Culture Collection
CAS	Chemical Abstracts Service
cDNA	Complementary DNA
CFU	Colony Forming Units
CLSI	Clinical and Laboratory Standards Institute
LM	*Listeria monocytogenes*
MIC	Minimum Inhibitory Concentration
PBS	Phosphate-Buffered Saline
RNA	Ribonucleic Acid
SD	Standard Deviation
TSA	Tryptic Soy Agar
TSB	Tryptic Soy Broth
