# Supplementary material for: Impact of Nisin on Growth Inhibition and Biofilm Formation Capacity of Listeria monocytogenes
Source: Int J Mol Sci. 2026 Aug 17;27(16):7355. doi: 10.3390/ijms27167355 (PMC13513324; doi:10.3390/ijms27167355)
Supplement: Supplementary file 1 [file ijms-27-07355-s001.zip › ijms-4447724-supplementary.pdf]

Table S1. One-way ANOVA testing for an overall treatment effect on each fitted parameter, within each strain (df = 6, 14; n = 3 replicates × 7 treatments).

Strain	Parameter	F	p-value	Significance
LM_ATCC 19115	Lag time (λ)	3.35	0.0291	*
	Max OD_{600}	12.81	<0.0001	****
	μ_{max}	0.64	0.6944	ns
LM_18	Lag time (λ)	53.68	<0.0001	****
	Max OD_{600}	16.01	<0.0001	****
	μ_{max}	11.12	0.0001	***

F, ANOVA test statistic; significance coding as in Table 1.

Table S2. Percentage of the 97 time points reaching significance vs. control using Tukey's HSD (Figure 2, uncorrected across time points).

Strain	Treatment	% significant - Tukey HSD
LM_ATCC 19115	1/8×MIC	1%
	1/4×MIC	3%
	1/2×MIC	37%
	1×MIC	35%
	2×MIC	99%
	4×MIC	99%
LM_18	1/8×MIC	33%
	1/4×MIC	37%
	1/2×MIC	47%
	1×MIC	74%
	2×MIC	100%
	4×MIC	100%

Table S3. RT-qPCR gene expression results (1/2×MIC, 6 h): each of three genes, in each strain, treated (nisin-exposed) vs. untreated control.

Strain	Gene	ΔΔCt	Fold change	Direction	p-value	Sig.
**LM_ATCC 19115**	*flaA*	-11.61	3118.6×	Up	0.0081	**
	hly	2.13	4.4× (down)	Down	0.2892	ns
	agrA	-8.72	421.7×	Up	0.0168	*
**LM_18**	*flaA*	-4.16	17.8×	Up	0.1806	ns
	hly	11.89	3786.5× (down)	Down	0.0251	*
	agrA	5.50	45.2× (down)	Down	0.0889	ns

*ΔΔCt and fold change are relative to that strain's own untreated control. Sig.: * p<0.05, ** p<0.01, ns = not significant (p≥0.05).*

Table S4. Primer sequences and optimal annealing temperatures for *Listeria monocytogenes* genes used in the RT-qPCR.

Gene	Primer seq (5′→ 3′)	Temperature (°C)
16S rRNA	F: AGCTTGCTCTTCCAAAGT<br>R: AAGCAGTTACTCTTATCCT	60
flaA	F: GACTTGTTACAAACAGCGGATTCA<br>R: ATTGACGCATACGTTGCAAGAT	60
agrA	F: GCAGCCGGACATGAATGG<br>R: AACCACGCGGATCAAACTTC	60
hly	F: TGACGAAATGGCTTACAGT	60
